# Blocking of Connexin-Mediated Communication Promotes Neuroprotection during Acute Degeneration Induced by Mechanical Trauma

**DOI:** 10.1371/journal.pone.0045449

**Published:** 2012-09-20

**Authors:** Vera Paschon, Guilherme Shigueto Vilar Higa, Rodrigo Ribeiro Resende, Luiz Roberto G. Britto, Alexandre Hiroaki Kihara

**Affiliations:** 1 Núcleo de Cognição e Sistemas Complexos, Centro de Matemática, Computação e Cognição, Universidade Federal do ABC, Santo André, São Paulo, Brazil; 2 Departamento de Fisiologia e Biofísica, Instituto de Ciências Biomédicas, Universidade de São Paulo, São Paulo, São Paulo, Brazil; 3 Departamento de Bioquímica e Imunologia, Universidade Federal de Minas Gerais, Belo Horizonte, Minas Gerais, Brazil; Universidad de Castilla-La Mancha, Spain

## Abstract

Accruing evidence indicates that connexin (Cx) channels in the gap junctions (GJ) are involved in neurodegeneration after injury. However, studies using KO animal models endowed apparently contradictory results in relation to the role of coupling in neuroprotection. We analyzed the role of Cx-mediated communication in a focal lesion induced by mechanical trauma of the retina, a model that allows spatial and temporal definition of the lesion with high reproducibility, permitting visualization of the focus, penumbra and adjacent areas. Cx36 and Cx43 exhibited distinct gene expression and protein levels throughout the neurodegeneration progress. Cx36 was observed close to TUNEL-positive nuclei, revealing the presence of this protein surrounding apoptotic cells. The functional role of cell coupling was assessed employing GJ blockers and openers combined with lactate dehydrogenase (LDH) assay, a direct method for evaluating cell death/viability. Carbenoxolone (CBX), a broad-spectrum GJ blocker, reduced LDH release after 4 hours, whereas quinine, a Cx36-channel specific blocker, decreased LDH release as early as 1 hour after lesion. Furthermore, analysis of dying cell distribution confirmed that the use of GJ blockers reduced apoptosis spread. Accordingly, blockade of GJ communication during neurodegeneration with quinine, but not CBX, caused downregulation of initial and effector caspases. To summarize, we observed specific changes in Cx gene expression and protein distribution during the progress of retinal degeneration, indicating the participation of these elements in acute neurodegeneration processes. More importantly, our results revealed that direct control of GJ channels permeability may take part in reliable neuroprotection strategies aimed to rapid, fast treatment of mechanical trauma in the retina.

## Introduction

Gap junction (GJ) channels are composed by two hexameric arrays of connexins (Cx), a multigene family of 20 integral membrane proteins described so far [1]. The association of six Cx makes a hemichannel (connexon), and the docking of two hemichannels in apposed cell membranes forms GJ channels, where gating and permeation properties differ depending on the Cx combination involved [2].

Communication mediated by GJ allows the transit of molecules up to 1 kDa between dying and viable neurons [3], although the results of this exchange, beneficial or detrimental, remain unclear. Interestingly, several studies have addressed this question using widespread apoptosis models, which do not create obvious molecular gradients between coupled cells. In these situations, experimental design may obscure whether spreading of apoptosis is actually related to GJ communication.

In order to settle this drawback, we have used an acute mechanical trauma model of the retina, which permits the visualization of the lesion focus, penumbra and adjacent areas. Moreover, in the retina ubiquitous cell coupling and high Cx expression were previously described. In this study, we focused on Cx36 and Cx43, which stand out as two of the most studied GJ proteins in the retina [4], [5].

**Figure 1:**
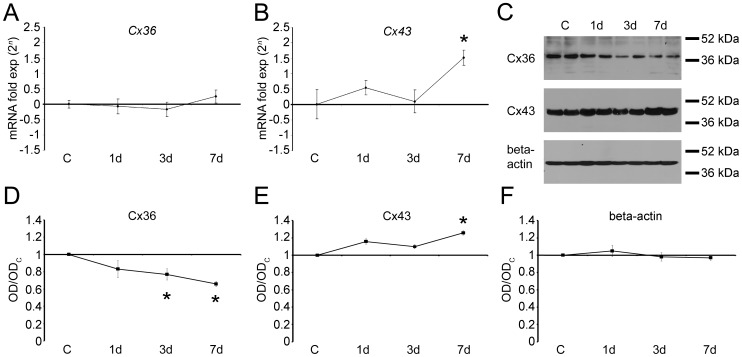
Connexin 36 (Cx36) and connexin 43 (Cx43) gene expression and protein levels after retinal neurodegeneration induced by mechanical trauma. We have analyzed *Cx36* and *Cx43* gene expression levels in the retina with quantitative real time PCR, using primers designed for chick *Cx36* and chick *Cx43*. Results were normalized by the mean of the control group, and were presented as fold-expression (2*^n^*). (A) We observed that *Cx36* gene expression levels were not significant altered after 1, 3 and 7 days post-lesion. (B) *Cx43* mRNA levels did not statistically change after 1 and 3 days. However, we observed a significant upregulation in the 7-day lesioned retinas (+182%, *P*<0.05). Protein levels were analyzed Western blot. Optical density (OD) from control retinas were used to normalize OD density from lesioned retinas in three independent experiments performed with duplicates. (C) Western blots of Cx36 and Cx43 in control and lesioned retinas using specific antibodies. Beta-actin (42 kDa) was used as an internal control. (D) We observed that Cx36 protein levels in the retina were downregulated after 3 (−23%, *P*<0.05) and 7 (−34%, *P*<0.05) days after the lesion. (E) On the other hand, Cx43 protein levels were upregulated after 7 days (+27%, *P*<0.05). Bars represent standard errors of mean. *P<0.05 *vs.* control in Newman-Keuls pairwise comparisons after one-way ANOVA.

## Methods

### Ethics Statement

These experiments were conducted in accordance with guidelines of the NIH and the Institute of Biomedical Sciences/USP.

### Animal Procedures

Experiments were carried out with *Gallus gallus*, with 7 to 15 days of age. Animals were anesthetized with ketamine/xylazine and submitted to 6 local mechanical lesions in the retina. Mechanical lesions were made with a thin needle (28-gauge, 12.7 mm, BD Diabetes, Franklin Lakes, NJ, USA). In some experiments, 20 µl of GJ blockers or PBS were injected right after the mechanical lesion. After different periods, the animals were killed with an overdose of ketamine (30 mg/100 g of body weight, i.m., Parke-Davis, Ann Arbor, MI, USA) and xylazine (2 mg/100 g, i.m., West Haven, CT, USA). The retinas were removed and dissected for different methodologies.

**Figure 2:**
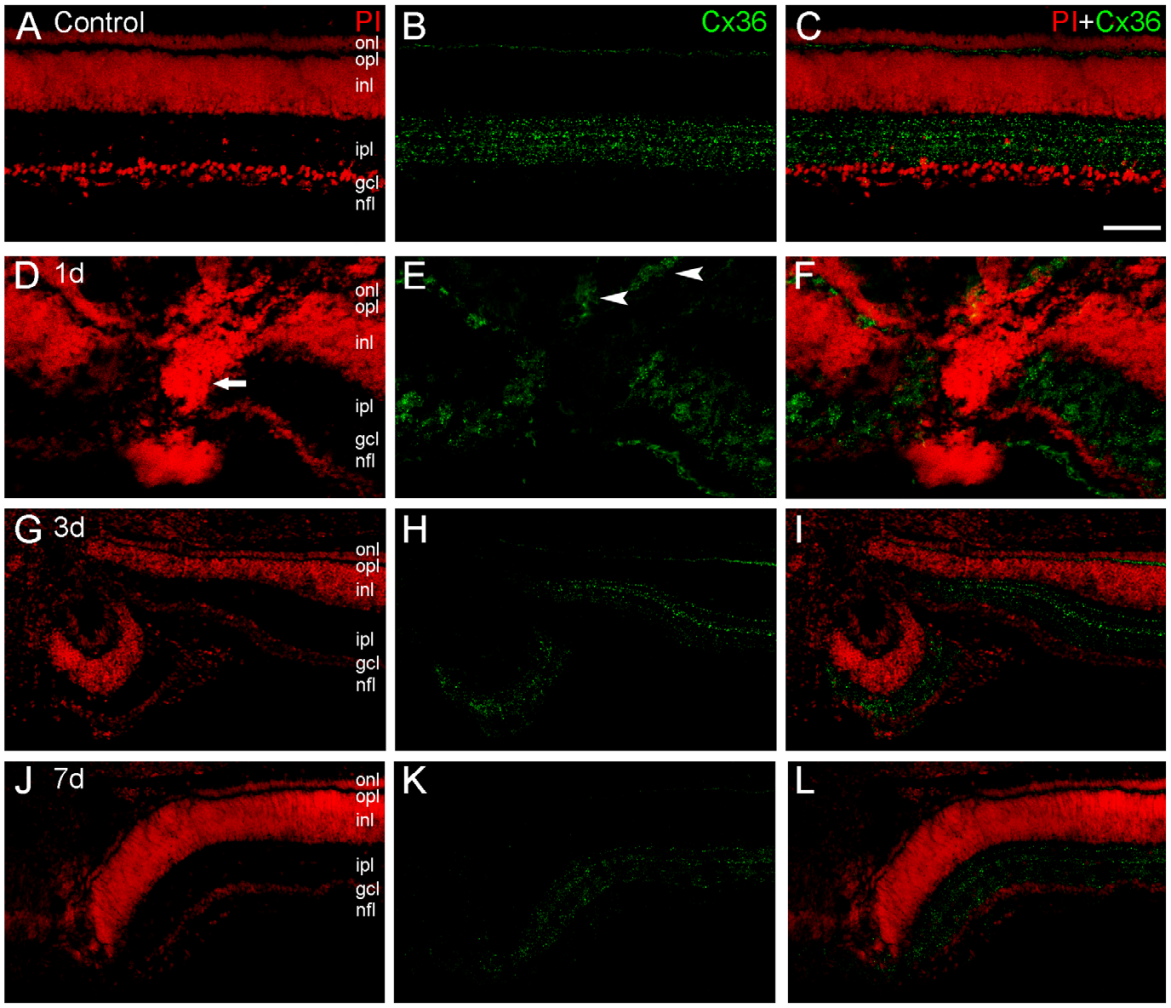
Connexin36 (Cx36) immunolabeling in transverse sections of the chick retina after mechanical lesions. We determined spatial distribution of Cx36 protein (green) in retinas counterstained with propidium iodide (PI, red) in control retinas (A–C) and after 1-(D–F), 3-(G–I) and 7-days (J–L) of the mechanical lesion. Control retinas showed a pattern of Cx36 distribution similar to that described in previous studies, with punctate labeling in the inner plexiform layer (IPL) forming horizontal lines, and in the outer plexiform layer (OPL) as well. In the lesion focus it is possible to observe an increase of PI labeling evidencing apoptotic nuclei (arrows). In 1-day lesioned retinas, an unexpected labeling pattern was observed in the outer nuclear layer (arrowheads), revealing that cells in this layer accumulate Cx36 protein close to the lesion. In the 3- and 7-day lesioned retinas, in spite the course of the degeneration process, Cx36 punctate labeling remains visible, indicating the presence of this protein after in these time points after the lesion. Scale bar: 60 µm.

### RNA Extraction, cDNA Synthesis and Real-time PCR

Retinas were directly homogenized in 1 ml TRIzol reagent (Invitrogen, Carlsbad, CA, USA) and total RNA was extracted following the manufacturer suggested protocol and previously described [6]. In brief, following two chloroform extraction steps, RNA was precipitated with isopropanol and the pellet washed twice in 70% ethanol. After air-drying, RNA was resuspended in DEPC-treated water and the concentration of each sample obtained from *A*_260_ measurements. Residual DNA was removed using DNase I (Amersham, Piscataway, NJ, USA) following manufacturer protocol. Quantitative analysis of Cx gene expression was carried out with a Rotor-Gene 6000 Real-Time Rotary Analyzer (Corbett Robotics Inc., San Francisco, CA) with specific primers for chick *Cx36* (forward, 5′-TTGGTGTTCATGTTTGCTGTCA-3′; reverse, 5′-CCAGCCCAAGTGGTTCAGTT-3′), chick *Cx43* (forward, 5′- CTGAGTGCCATCTACACCTGTGA-3′; reverse, 5′-TTGGACGGGACAGGAAACA-3′), chick *caspase 3* (forward, 5′- CCGGACTGTCATCTCGTTCA-3′; reverse, 5′- TTTATATCCCAGCTTCATAAAAACTTCTC-3′), chick *caspase 8* (forward, 5′- TGGGCAGGAAGTACCTATCCA-3′; reverse, 5′- CCAGCAAGTGATTGGCAATTT-3′) and chick *caspase 9* (forward, 5′- ACCTTGGACAGCGTACTGGAA-3′; reverse, 5′- TGACACCCGAAGTAGCTTGGT-3′). cDNA abundance for *GAPDH* was also determined as an internal control (forward, 5′-GGTGCTAAGCGTGTTATCATCTCA-3′; reverse, 5′-CATGGTTGACACCCATCACAA-3′). For each 20 µl reverse transcription reaction, 4 µg total RNA was mixed with 1 µl oligo dT primer (0.5 µg; Invitrogen) and incubated for 10 min at 65°C. After cooling on ice the solution was mixed with 4 µl 5× first strand buffer, 2 µl of 0.1 M DTT, 1 µl of dATP, dTTP, dCTP and dGTP (each 10 mM), and 1 µl SuperScript III reverse transcriptase (200 U; Invitrogen) and incubated for 60 min at 50°C. Reaction was inactivated by heating at 70°C for 15 min. All PCR assays were performed as follows: after initial activation at 50°C for 2 min and 95°C for 10 min, cycling conditions were 95°C, 10 s and 60°C, 1 min. Dissociation curves of PCR products were obtained by heating samples from 60°C to 95°C, in order to evaluate primer specificity.

### PCR Statistical Analysis

Relative quantification of target gene expression was evaluated using the comparative CT method as previously described in detail [7]. Fold-changes in gene expression of the target gene are equivalent to 2^−ΔΔCT^. Values were entered into a one-way analysis of variance (ANOVA), followed by pairwise comparisons (Tukey’s HSD test), with the significance level set at 5%.

**Figure 3:**
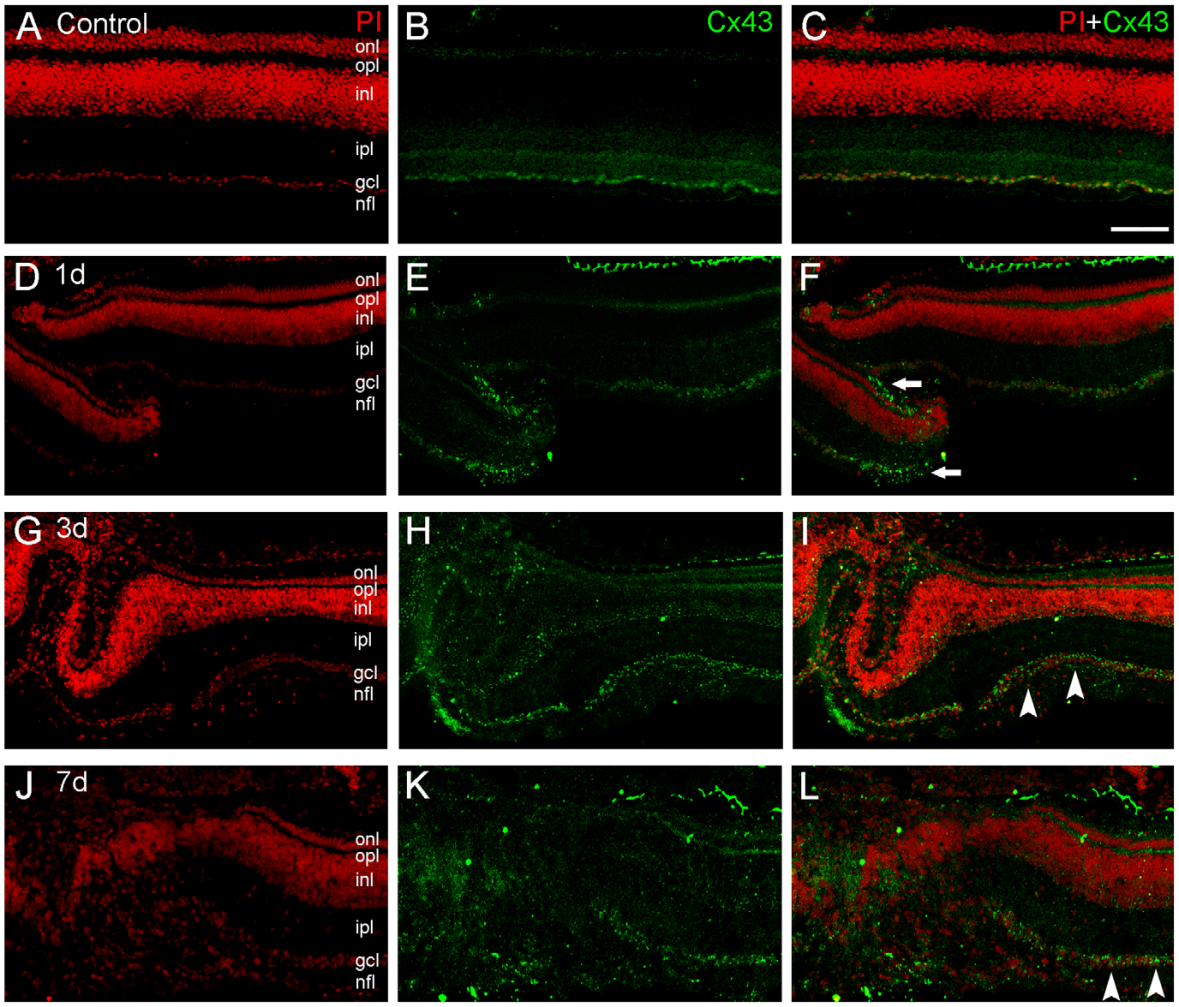
Connexin43 (Cx43) immunolabeling in transverse sections of the chick retina after mechanical lesions. We determined spatial distribution of Cx43 protein (green) in retinas counterstained with propidium iodide (PI, red) in control retinas(A–C) and after 1- (D–F), 3- (G–I) and 7-days (J–L) of the mechanical lesion. Control retinas showed a pattern of Cx43 distribution similar to the that described in previous studies, with punctate labeling located in the ganglion cell layer (GCL), and fine punctate labeling located in the inner plexiform layer (IPL) and in the outer plexiform layer (OPL). In 1-day lesioned retinas, we observed an increased labeling for Cx43 in the focus of the lesion. Outsized and brighter punctate labeling was seen in the ganglion cell layer, surrounding large nuclei of presumptive ganglion cells (arrows). Notice that some puncta located in the center of the image correspond to labeling in the GCL. In addition, in the upper part of the image, Cx43 labeling was seen in the pigmented epithelium, as described in previous studies. After 3 days, Cx43 punctate labeling was seen in the vicinity of the lesion focus, in the penumbra area, within the GCL (arrowheads), and also in the inner part of the inner nuclear layer. In 7-day lesioned retinas, we observed a dusty punctate labeling in the focus of the lesion, with a disorganized pattern. Away from the focus, the typical connexin punctate immunolabeling was seen in the GCL. Scale bar: 60 µm.

### Western Blotting

As previously described [8], retinas were rapidly dissected, washed with phosphate buffered saline (PBS), and homogenized in 25 mM Tris buffer (Calbiochem, La Jolla, CA, USA). Homogenates were centrifuged for 5 min at 2000 G to remove insoluble material. Protein concentration was determined by the Bradford method and bovine serum albumin was used as the standard. Proteins in the membrane preparations were separated by sodium dodecyl sulfate-polyacrylamide gel electrophoresis (SDS-PAGE; 4–20% gradient gel) and transferred to nitrocellulose membranes. Blots were incubated with 5% milk in PBS with 0.2% Tween-20 for 1 h at room temperature to block nonspecific binding of the antibodies. After rinsed in PBS, blots were incubated overnight with primary antibodies raised against Cx36 (1∶250) and Cx43 (1∶1000) and beta-actin (1∶1000, Sigma-Aldrich, St. Louis, MO, USA, catalog # A5316) diluted in PBS/0.2% Tween-20/1%milk. After the primary antibody incubation, blots were rinsed in PBS/0.2% Tween-20/5% milk and incubated with goat anti-rabbit-peroxidase (ECLTM kit; Amersham, Buckinghamshire, England) for 2 h at room temperature. Detection of labeled proteins was achieved by using the enhanced chemiluminescent system (ECLTM kit; Amersham). Measurements of optical densities were performed using Scion Image software (Scion Corporation, Frederick, Maryland, USA). Optical densities (OD) of the bands were normalized using the value found for the adult. Data from three independent experiments were entered into a one-way ANOVA, using repeated measures design, followed by pairwise comparisons with Tukey’s HSD test.

### Immunohistochemistry

Eyes were dissected out and the retinas were fixed for 30 minutes in 1% paraformaldehyde (PFA) in phosphate buffer 0.1 M pH 7.3 (PB), and cryoprotected in 30% sucrose solution for at least 24 hours at 4°C. After embedding in O.C.T. compound (Sakura Finetek, Torrance, CA, USA) they were cut transversally (12 µm) on a cryostat. Retinal sections were blocked for 30 minutes in a solution containing 10% normal goat serum, 1% bovine serum albumin (BSA) and 0.5% Triton–X 100 in PBS.

**Figure 4:**
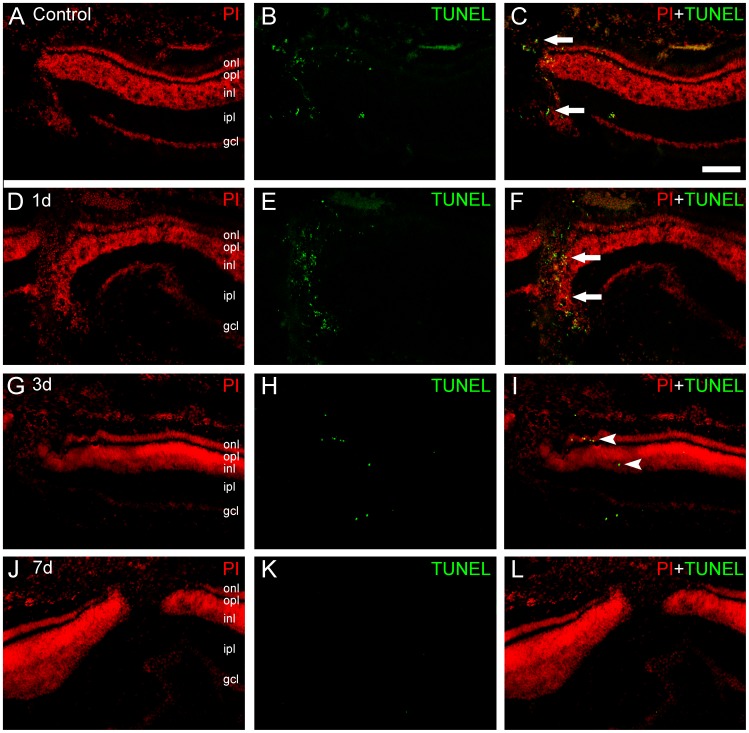
TUNEL labeling in retinas after mechanical lesions. Transverse sections of lesioned chick retinas were submitted to terminal deoxynucleotidyl transferase dUTP nick end labeling (TUNEL, green) to characterize the apoptosis spatial pattern, and counterstained with propidium iodide (PI, red). A–C, after 6 h of the mechanical trauma, we could observe TUNEL-positive nuclei in the focus of the lesion (arrows). D–F, in 24 h-lesioned retinas, we observed a peak in the number of the TUNEL-positive cells, mainly located in the focus of the lesion. These cells were mainly distributed in the inner nuclear layer (INL), but also in the outer nuclear layer (ONL). Notice that some cells located in the lower part of the image were actually from the INL and displaced by the mechanical trauma. G–I, after 3 days, we observed a decrease in the number of TUNEL-positive cells. Moreover, these cells were located away from the focus, in the penumbra area (arrowheads), evidencing the secondary cell death. In 7-day lesioned retinas, we typically observed very few or no TUNEL-positive cells in retinal transverse sections. Scale bar: 60 µm.

We previously sequenced the immunogenic peptide of the anti-Cx36 antibody used in the present study [9], a polyclonal antibody raised against amino acids 296–304 of human Cx36 (1∶100–1∶200, Zymed/Invitrogen, catalog #36–4600). This immunogenic peptide shares 100% homology with chick Cx36/35.1 sequence in the carboxyl terminus. The specificity of this antibody was previously tested in retinas of Cx36−/− KO mice [9] and was previously employed in chick retinal studies [10]. To identify Cx43, we used a polyclonal antibody against amino acids 251–270 of rat Cx43 (1∶200, #71–0700, Zymed/Invitrogen). This amino acid sequence shares 100% homology with chick Cx43 and was previously used in chick retinal studies [11]. We also used a polyclonal antibody to detect choline acetyltransferase (ChAT, 1∶200, #AB144P, Merck-Millipore, Billerica, MA, USA), aimed at identifying a specific class of retinal neurons.

After several washes, retinal sections were incubated with goat antiserum against rabbit or mouse IgG tagged to Alexa TM 488 (Molecular Probes, Eugene, Oregon, 1∶250–1∶500) diluted in PBS containing 0.5% Triton X-100 for 2 hours at room temperature. We used secondary antibodies conjugated to fluorescein isothiocyanate (FITC, Jackson Labs, West Grove, PA, USA, 1∶250–1∶500), to tetramethyl rhodamine isothiocyanate (TRITC, Jackson Labs, 1∶250–1∶500) and to indodicarbocyanine (Cy5, Jackson Labs, 1∶250–1∶500). Controls for the experiments consisted of the omission of primary antibodies; no staining was observed in these cases. After washing, the tissue was mounted using Vecta Shield (Vector Labs, Burlingame, CA, USA), and analyzed in a Nikon PCM2000 confocal microscope. Figures were mounted with Adobe Photoshop CS. Manipulation of the images was restricted to threshold and brightness adjustments to the whole image.

**Figure 5:**
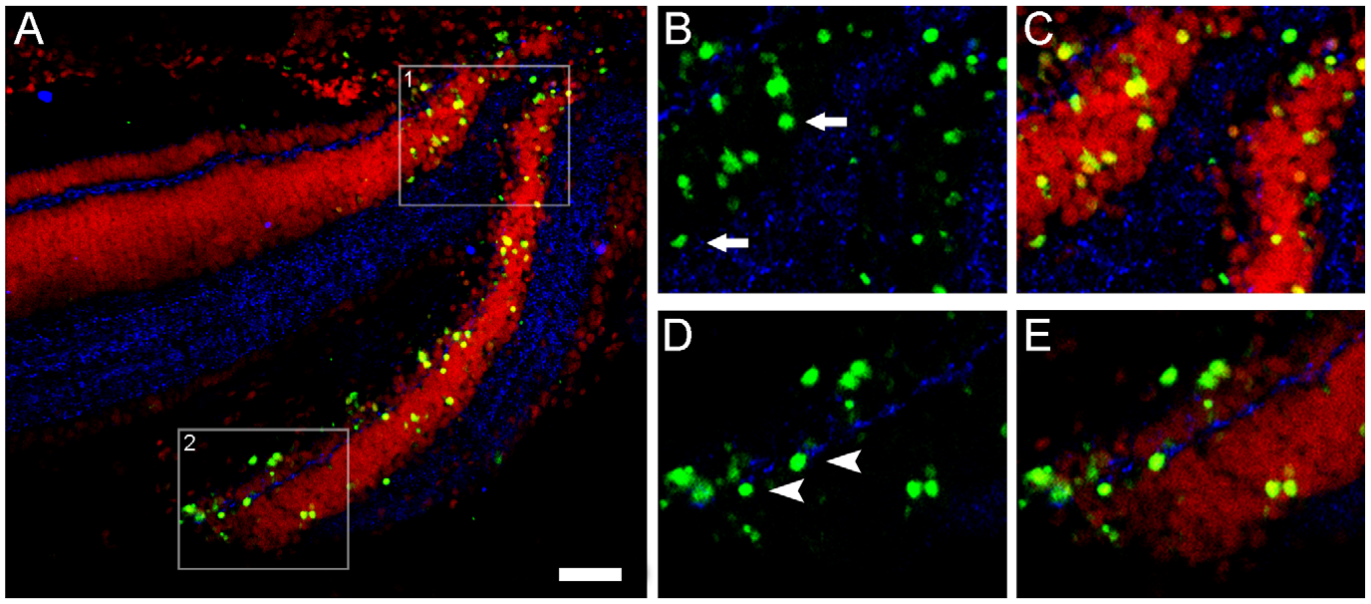
Connexin36 (Cx36) and TUNEL- positive nuclei spatial relationship within the focus and penumbra of the lesion. In order to determine the spatial relationship between Cx36 and TUNEL-positive nuclei, TUNEL assay (green) was carried out with Cx36 immunofluorescence (blue) and propidium iodide counterstaining (PI, red) in transverse sections of 1-day lesioned chick retinas. (A) We typically observed TUNEL-positive nuclei close to Cx36 punctate labeling, indicating that apoptotic cells may accumulate Cx36 protein. With high magnification of selected areas 1 and 2, we observed the close spatial relationship between Cx36 puncta and TUNEL-positive nuclei. (B, C) We detected several TUNEL-positive nuclei located in the outer nuclear layer (arrows) surrounded by Cx36 puncta. (D, E) In addition, we observed TUNEL-positive nuclei throughout the inner nuclear layer, including cells located in the outer margin (arrowheads), but in the inner margin as well. This spatial pattern revealed that different retinal cell types express Cx36 during apoptosis. Scale bar: 60 µm.

### Terminal Deoxynucleotidyl TRANSFERASE (TdT)-Mediated dUTP Nick-End Labeling (TUNEL) Assay

As previously described [12], 12 µm cryostat sections from lesioned sites or corresponding locations in control eyes were fixed in 4% PFA for 60 min at 4°C. Labeling was accomplished by incubation in TdT buffer for 10 min at room temperature followed by incubation with a biotinylated 2-deoxy-uridine- 5-triphosphate (dUTP) (Roche Molecular Biochemicals, Mannheim, Germany). The reaction was terminated in stop reaction buffer (10%, 0.1 M ethylenediaminetetraacetic acid, pH 8, in water) and washed in PBS. The sections were mounted using the ProLong Antifade kit (Molecular Probes).

### Retinal Explants and Lactate Dehydrogenase Assay (LDH)

Animals with 24 hour lesioned retinas were sacrificed and retinas were conditioned in culture medium for 4 hours at room temperature. Medium was prepared with basal medium of eagle (BME), 5% bovine fetal serum, 1% glutamine and GJ blockers or openers diluted in PBS (50 or 100 µM). After 1, 2 and 4 hours, 400 µl of the culture medium were collected and centrifuged at 100 g for 5 min.

Quantification of lactate dehydrogenase (LDH) released into the culture medium is used as an indicator of cell viability [13]. In our experiments, cell viability was assessed by measuring LDH released in the explant medium, expressed as a percentage of total LDH released by the retina [14]. Drugs that affect GJ permeability used in this study were i) carbenoxolone (CBX), a broad-spectrum GJ blocker, ii) quinine, a Cx36- and Cx50-specific blocker, and iii) trimethylamine hydrochloride (TMA), a GJ opener. LDH was measured from aliquots of supernatant with colorimetric quantification a using specific biomedical kit (Labtest Diagnostica, Lagoa Santa, MG, Brazil). Results were obtained by measuring light absorbance at 500 nm.

**Figure 6:**
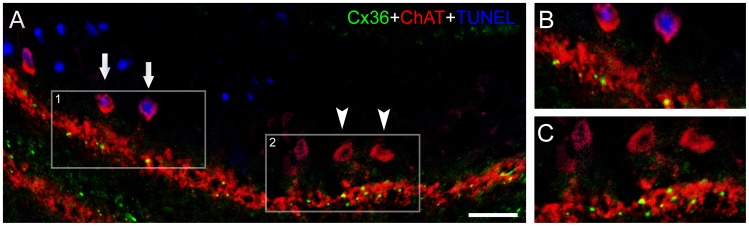
Presence of connexin36 (Cx36) in specific apoptotic neurons. (A) In order to confirm the presence of Cx36 in specific apoptotic neurons, we performed triple-labeling experiments to simultaneously localize Cx36 (green), choline acetyltransferase (ChAT, red) and TUNEL-positive nuclei (blue) in transverse sections of 1-day lesioned chick retinas. With high magnification of selected areas 1 and 2, it is possible to observe colocalization of Cx36 and processes of ChAT-positive cells. (B) Indeed, Cx36 typical punctate labeling was seen in processes of apoptotic cholinergic amacrine cells (arrows). (C) As expected, Cx36 punctate labeling was also observed in processes of non-apoptotic ChAT-positive cells (arrowheads). These results confirmed that at least the cholinergic amacrine cells maintain the accumulation of Cx36 during apoptosis. Scale bar: 60 µm.

### Image Quantification

Image analyses were performed with Image-Pro Plus software (Media Cybernetics, Bethesda, MD, USA). After channel separation (RGB) of color images, we performed two different analyses: i) Counting cells/nuclei: after proper setting of size and brightness, the software performs automatic search of discrete elements, as punctate labeling. Automatically counted labeling was displayed in the image, thus artifacts and background labeling could be identified and discarded. ii) Bitmap analysis: *x*–*y* axis analyses generated numerical appended data file corresponding to pixel values. The bitmap analysis was used to view the pixel values of the active window (or area of interest, AOI) in numeric format, where values correspond to the brightness of the pixels. This matrix was exported to Excel (Microsoft, Redmond, WA, USA) for the appropriate mathematical computation. The numerical data generated a histogram, essentially averaging the labeling intensity at different retinal locations. Photomicrographs and charts were prepared using Adobe Photoshop CS2 (Adobe Systems Inc., San Jose, CA, USA).

**Figure 7:**
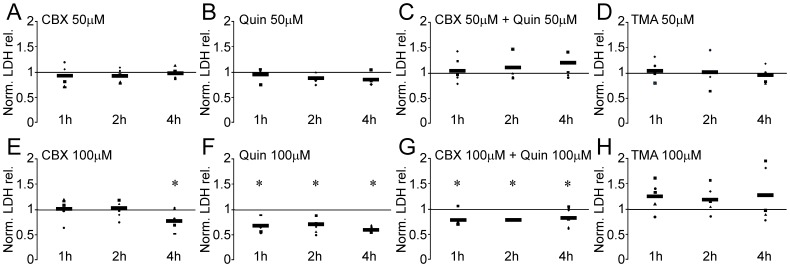
Assessment of cell viability using quantification of lactate dehydrogenase (LDH) released by lesioned retinas treated with GJ blockers and openers. Retinas were removed after 1 day of binocular mechanical trauma. One retina was conditioned in medium containing PBS (control) whereas the other was conditioned with pharmacological agents (experimental), which were described to modulate cell coupling, either closing (cabenoxolone, CBX; quinine, Quin) or opening (trimethylamine hydrochloride, TMA) the GJ channels. LDH in the retinal explant medium was measured after 1, 2 and 4 hours of in vitro treatment, using the appropriate kit followed by quantification by spectrophotometric analysis. “Experimental” dots were shown as LDH quantification normalized by the respective control, with the horizontal bars representing the means. In some cases, experimental dots overlapped each other and/or the horizontal bar. We have tested two different concentrations of the pharmacological agents, i.e. 50 and 100 µM. We observed no changes in LDH release in retinal explants treated with (A) CBX 50 µM, (B) Quin 50 µM, (C) CBX+Quin 50 µM and (D) TMA 50 µM. On the other hand, we observed changes in LDH release when retinas were treated with the same drugs with a higher concentration (100 µM). (E) After incubating with CBX 100 µM for 4 h, we detected a significant decrease in the LDH release (−22%, *P*<0.01). (F) Incubation with Quin 100 µM decreased LDH release after 1 h (−31%, *P*<0.01), 2 h (−28%, *P*<0.01) and 4 h (39%, *P*<0.01). (G) CBX 100 µM and quinine 100 µM produced similar results: LDH release decreased after 1 h (−21%, *P*<0.01), 2 h (−21%, *P*<0.01) and 4 h (−16%, *P*<0.01) when compared to controls. (H) We observed no significant changes in LDH release using TMA 100 µM in any of the analyzed time points. *P<0.01 in paired T Test (n = 5).

## Results

### Cx Gene Expression and Protein Levels Are Specifically Regulated during Retinal Degeneration

We were able to detect *Cx36* and *Cx43* gene transcripts by using real time PCR in the proposed model of neurodegeneration in different periods after mechanical lesion (1, 3 and 7 days). Specificity of chick Cx36 and Cx43 primers were attested in previous studies [10], [11] and by analysis of dissociation curves (data not shown).

*Cx36* and *Cx43* have distinct expression profiles during retinal degeneration induced by mechanical trauma. *Cx36* gene expression was not significant regulated in any of the analyzed periods (Fig. 1A). On the other hand (Fig. 1B), we observed that *Cx43* mRNA levels slightly increased after 1 day, returned approximately to the steady-state levels after 3 days and was significantly up regulated after 7 days (182%, *P*<0.05).

**Figure 8:**
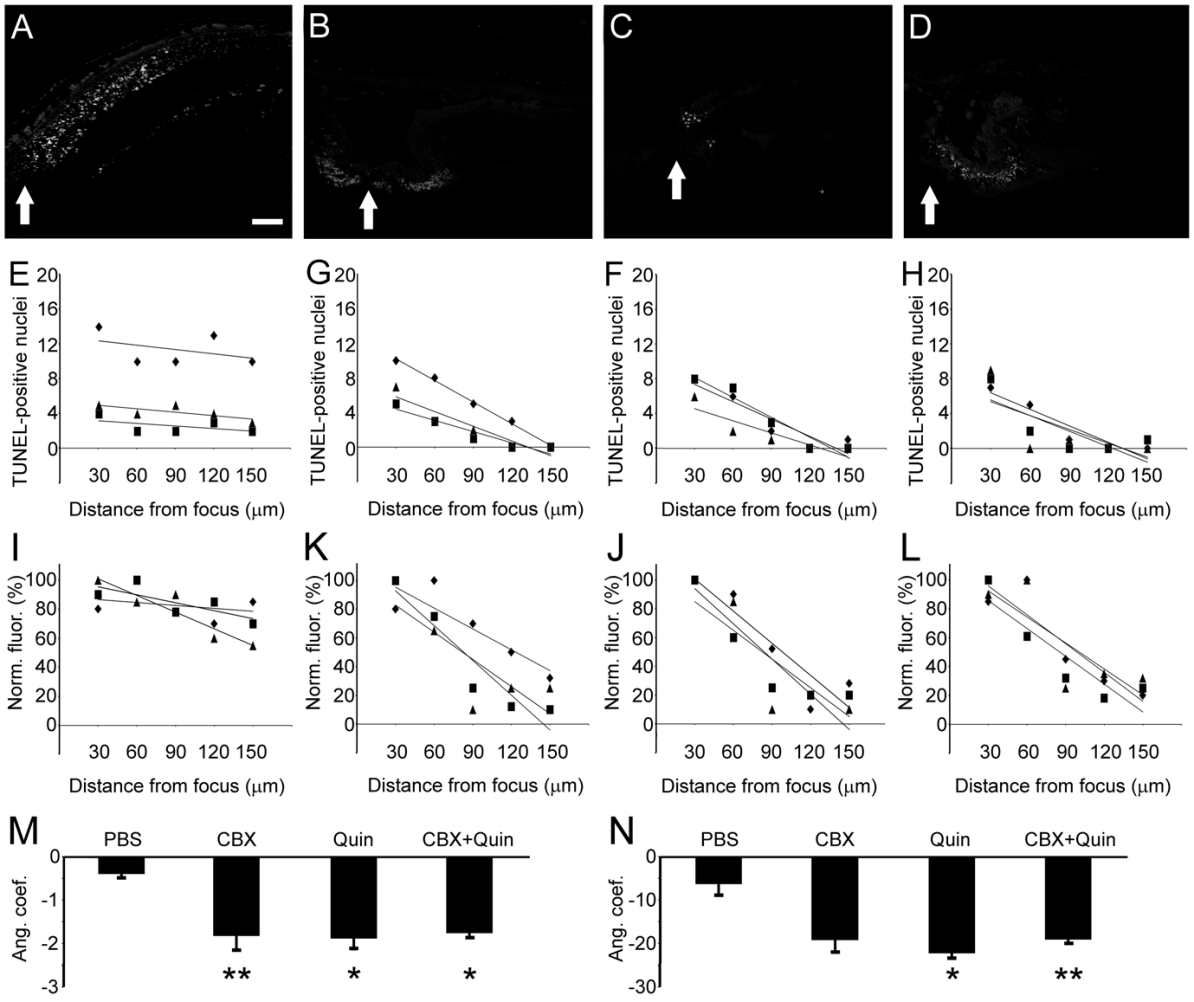
TUNEL labeling in retinal explants treated with GJ blockers. Retinal explants were cultured for 4 hours with (A) PBS, (B) carbenoxolone (CBX), (C) quinine (Quin) and (D) CBX 100 µM + quinine 100 µM. Transverse sections of chick retina explants were submitted to terminal deoxynucleotidyl transferase dUTP nick end labeling (TUNEL) to characterize apoptotic spatial pattern. In each image it is possible to localize the focus of the lesion (arrows). (E–H) In order to determine whether GJ blockers caused changes in the distribution of apoptotic cells, we have counted the number of TUNEL- positive nuclei located as far as 150 µm away from the focus of the lesion. Values were plotted according to the distance of the focus, and were submitted to linear regression using the least square approach, generating mathematical parameters such as *R^2^*, *R* and also first order equation (*y  =  ax + b*). (I–L) The same procedure was undertaken using values from pixel analysis. *X–Y* axis bitmap analysis was used to view the pixel values in numeric format, where values correspond to the brightness of the pixels. (M) Considering the distribution of TUNEL-positive nuclei, the means of angular coefficient from first order equation (*a*) were calculated for each experimental condition (n = 3). When compared to the control (PBS), the means of *a* angular coefficient were higher for all evaluated conditions using GJ blockers (CBX, quinine and CBX + quinine). (N) Regarding values from bitmap pixel analysis, we observed that the means of *a* angular coefficient were higher for quinine and CBX + quinine conditions. *P<0.01 and **P<0.05 vs. PBS in Newman-Keuls pairwise comparisons after one-way ANOVA. Scale bar: 60 µm.

We also analyzed Cx36 and Cx43 protein levels in western blots (Fig. 1C). Cx36 significantly decreased after 3 (−23%, *P*<0.05) and 7 (−34%, *P*<0.05) days after the lesion (Fig. 1D). On the other hand, we observed a significant increase (+27%, *P*<0.05) of Cx43 after 7 days (Fig. 1E). Beta-actin blots were used as internal control (Fig. 1F).

### Cx36 Maintains Ubiquitous Distribution during Retinal Degeneration

We analyzed Cx36 spatial pattern during retinal degeneration caused by mechanical lesion. Control retinas showed a spatial distribution similar to the pattern described in previous studies [10], with punctate labeling in the inner plexiform layer, forming horizontal lines. In addition, punctate labeling was seen in the outer plexiform layer (Fig. 2, A–C). After 1-day post-lesion, an unexpected labeling pattern was observed in the outer nuclear layer, namely the occurrence of cells accumulating Cx36 protein close to the lesion site (Fig. 2, D–F). In 3-day lesioned retinas (Fig. 2, G–I), punctate labeling was seen close the lesion focus. After 7 days (Fig. 2, J–L), in spite the course of the degeneration process, Cx36 punctate labeling remained visible, indicating the presence of this protein at this time.

**Figure 9:**
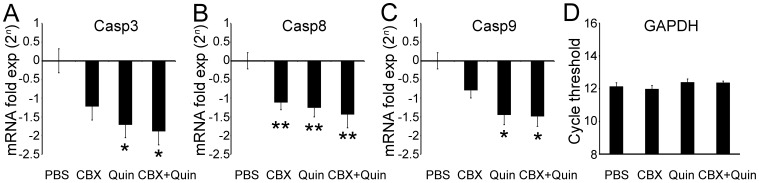
Caspase gene expression in retinal explants treated with GJ blockers. One-day lesioned retinal explants were cultured for 4 hours with PBS, carbenoxolone 100 µM (CBX), quinine 100 µM (Quin) and CBX 100 µM + Quin 100 µM. (A) We observed downregulation of *caspase 3* gene expression in retinal explants cultured with Quin (−69%, *P*<0.01) and CBX+Quin (−73%, *P*<0.01). (B) On the other hand, *caspase 8* expression was downregulated after CBX (−54%, *P*<0.05), Quin (−58%, *P*<0.05) and CBX + Quin (−63%, *P*<0.05) treatment. (C) Similar to *caspase 3*, *caspase 9* gene expression was downregulated with Quin (−63%, *P*<0.01) and CBX + Quin (−64%, *P*<0.01). All caspase gene expression results were normalized by the mean of the PBS group, and were presented as fold-expression (2*^n^*). (D) *GAPDH* abundance was used as internal control. Bars represent standard errors of the means. *P<0.01 and **P<0.05 vs. PBS in Newman-Keuls pairwise comparisons after one-way ANOVA.

### Cx43 Is Mainly Observed Among Ganglion Cells during Retinal Degeneration

We also determined spatial distribution of Cx43 in the retina after the mechanical lesion. Control retinas showed an spatial pattern distribution similar to the pattern described in previous studies [11], with clear punctate labeling located in the ganglion cell layer, plus small, dusty punctate labeling located in the inner plexiform layer and in the outer plexiform layer, as well (Fig. 3, A–C). In 1-day lesioned retinas, we observed an increased labeling for Cx43 in the focus of the lesion. Outsized and brighter punctate labeling was seen in the ganglion cell layer, surrounding large nuclei of presumptive ganglion cells. In addition, Cx43 labeling was seen in the pigmented epithelium, as described in previous studies (Fig. 3, D–F). After 3 days, Cx43 punctate labeling was seen in the penumbra area, within the ganglion cell layer, and also in the inner part of the inner nuclear layer as well (Fig. 3, G–I). In 7-day lesioned retinas, we observed a dusty punctate labeling in the focus of the lesion, with a disorganized pattern (Fig. 3, J–L).

**Figure 10:**
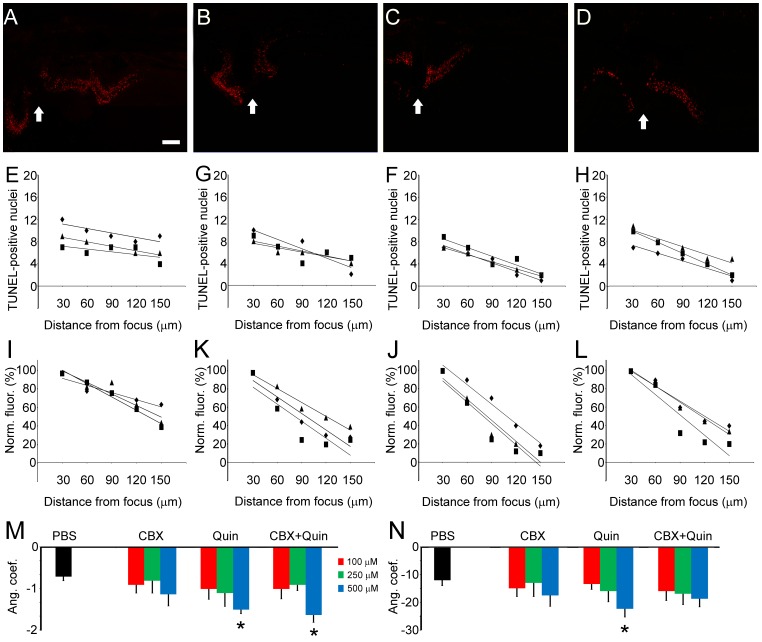
TUNEL labeling in retinas from eyes injected with GJ blockers. Right after mechanical trauma in the retina, eyes were injected with (A) PBS, (B) carbenoxolone (CBX), (C) quinine (Quin) and (D) CBX 500 µM + Quin 500 µM. Animals were euthanized 1 day post-lesion, and transverse sections of retinas were submitted to terminal deoxynucleotidyl transferase dUTP nick end labeling (TUNEL) to characterize apoptotic spatial pattern. In each image it is possible to localize the focus of the lesion (arrows). (E–H) In order to determine whether GJ blockers caused changes in the distribution of apoptotic cells, we have counted the number of TUNEL- positive nuclei located as far as 150 µm away from the focus of the lesion. Values were plotted according to the distance of the focus, and were submitted to linear regression using the least square approach, generating mathematical parameters such as *R^2^*, *R* and also first order equation (*y  =  ax + b*). (I–L) The same procedure was undertaken using values from pixel analysis. (M) Considering the distribution of TUNEL-positive nuclei, the means of angular coefficient from first order equation (*a*) were calculated for each experimental condition (n = 3). When compared to the control (PBS), the means of *a* angular coefficient were higher for Quin 500 µM and CBX 500 µM + Quin 500 µM. (N) Regarding values from bitmap pixel analysis, we observed that the mean of *a* angular coefficient was higher for retinas from eyes injected with Quin 500 µM. *P<0.05 vs. PBS in Newman-Keuls pairwise comparisons after two-way ANOVA. Scale bar: 60 µm.

### Peak of Apoptosis Occurs between 6 and 24 Hours After the Mechanical Trauma

Experiments using the TUNEL technique were carried out in other to determine the dynamics of the apoptotic process. We were able to detect apoptotic nuclei in retinas as early as 6 hours after the onset of the mechanical trauma (Fig. 4, A–C), which were located mainly in the focus of the lesion.

In 24 h-lesioned retinas, we observed a peak in the number of the TUNEL-positive cells, mainly located in the focus of the lesion (Fig. 4, D–F). These cells were mainly distributed in the inner nuclear layer, but also occurred in the outer nuclear layer. After 3 days, we observed a decrease in the number of the TUNEL-positive cells. These cells were located away from the focus, in the penumbra area, evidencing the secondary cell death (Fig. 4, G–I). In 7-day lesioned retinas, we typically observed very few, or often none, TUNEL-positive cells in retinal transverse sections (Fig. 4, J–L). Taken together, these results evidence the presence of apoptotic cells in the neighborhood of the injury in the analyzed periods. Moreover, our results disclose that apoptotic nuclei are mainly located in the inner and outer nuclear layers.

### Cx36 Occurs Close to Apoptotic Cells

Our results indicated that the peak of cell death occurs 1 day after the injury, and that apoptotic cells were concentrated in the inner and outer nuclear layers. Combination of these data with distribution of the analyzed Cx indicated that Cx36, but not Cx43, could most likely be involved in the apoptosis of retinal cells. Thus, we carried out experiments to investigate whether apoptotic cells express Cx36. In several instances, we detected TUNEL-positive nuclei close to Cx36 punctate labeling, indicating that apoptotic cells may accumulate Cx36 protein (Fig. 5A). With high magnification of specific areas, we could observe a close spatial relationship between Cx36 puncta and TUNEL-positive nuclei (Fig. 5, B–E). We detected several TUNEL-positive nuclei located in the outer nuclear layer surrounded by Cx36 puncta. We also observed TUNEL-positive nuclei throughout the inner nuclear layer, including cells located both in the inner and the outer margins of this layer. This spatial pattern disclosed that different retinal cell types express Cx36 during apoptosis.

### Amacrine Cells Maintain the Expression of Cx36 during Apoptosis

Once we detected TUNEL-positive nuclei located in the inner nuclear layer, we performed triple-labeling experiments to determine whether dying neurons maintain the expression of Cx36. For this purpose, we employed antibody raised against choline acetyltransferase (ChAT), which is an enzyme found in cholinergic neurons in the central nervous system.

As shown in Figure 6, Cx36 displayed the usual punctate labeling. In the chick retina, anti- ChAT labels two distinct cholinergic amacrine cells populations: 1) amacrine cells with somata located in the inner nuclear layer, whose processes arborize on sublayer 2 of the inner plexiform layer, and 2) displaced amacrine cells with somata located in the ganglion cell layer, whose processes arborize on sublayer 4 of the inner plexiform layer. Indeed, we previously observed that Cx36 accumulate in processes of ChAT amacrine cells 10. Interestingly, in this study the presence of Cx36 was observed in apoptotic ChAT-positive cells. These results confirmed that at least a specific population of amacrine cells maintains the expression of Cx36 during apoptosis.

### GJ Blockers Increase Cell Viability in Retinal Explants

In addition to determine changes in Cx expression and distribution, we also approached to the functional role of Cx-mediated communication in this retinal degeneration model. For this purpose, we assessed the effects of GJ blockers (CBX and quinine) and opener (TMA) in retinal explants [15], [16]. After 1 day of the mechanical trauma, retinas were cultured in proper medium and samples were collected after 1, 2 and 4 hours. All assays that were carried out with lower concentrations (50 µM) resulted in no significant changes in LDH release (Fig. 7, A–D). On the other hand, the use of GJ blockers at 100 µM decreased LDH release in the explant medium. Incubation of retinas with the broad spectrum GJ blocker CBX (Fig. 7E) for 4 hours caused significant decrease (−22.1%, P<0.01) in LDH release. In experiments carried out with quinine (Fig. 7F), a specific Cx36 and Cx50 channel blocker, we observed an earlier, immediate effect. Retinas incubated with quinine released less LDH than controls after 1 (−30.6%, P<0.01), 2 (−27.9, P<0.01) and 4 hours (−38.9%, P<0.01). The combination of CBX and quinine (Fig. 7G) also resulted in a decrease in LDH release at all analyzed periods, 1 (−21.1%, P<0.01), 2 (−20.9%, P<0.01) and 4 hours (−16.1%, P<0.01). On the other hand, experiments with the GJ opener TMA resulted in no significant changes in LDH released (Fig. 7H).

### GJ Blockers Limit Apoptosis Spread *In Vitro*

Since our results indicated that the use of GJ blockers increase cell viability in lesioned retinas, we further investigated whether these blockers endow changes in the distribution of apoptotic cells. Thus, 1-day lesioned retinas were kept in culture for 4 hours with PBS and GJ blockers. After this period, transverse sections of retinal explants were obtained for TUNEL labeling.

Distribution of apoptotic cells changes in retinal explants treated with GJ blockers. Taking the lesion focus as a reference, distribution of apoptotic nuclei was typically narrower in retinas incubated with GJ blockers (Fig. 8, A–D), although some variations in the spatial distribution were also observed. In order to test the consistence of this result, we employed two different quantification methods. First, we plotted the number of apoptotic nuclei counted from different distances from the lesion focus. Next, data plots were submitted to a linear regression using least square approach, generating parameters such as *R^2^*, *R* and also first order equation (*y  =  ax + b*) for each experimental condition (Fig. 8, E–H). As a second approach, values from bitmap analyses were submitted to the same procedure (Fig. 8, I–L). The bitmap analysis was used to view the pixel values of active windows in numeric format, where values correspond to the brightness of the pixels.

The means of angular coefficient from first order equation (*a*) was calculated for each experimental condition (n = 3), as a parameter of apoptosis spread. Regarding the number of apoptotic cells (Fig. 8M), the absolute value for *a* angular coefficient was higher for all evaluated conditions using GJ blockers: CBX (−1.83±0.35, P<0.05), quinine (−1.90±0.26, P<0.01) and CBX + quinine (−1.77±0.09, P<0.01), when compared to PBS (−0.40±0.06). Likewise, values obtained from bitmap pixel analysis (Fig.8N) indicated that the decrease of normalized pixel brightness is larger when retinas were maintained in quinine (−22.30±1.30, P<0.01) or CBX + quinine (−19.13±0.55, P<0.05), when compared to PBS (−6.33±2.77). Taken together, these results revealed that apoptosis spread is reduced when retinas are treated with GJ blockers.

### GJ Blockers Downregulate Caspase Gene Expression during Retinal Degeneration

Considering the evidences provided by our in vitro findings, we investigated the effects of GJ blockers these drugs on caspase gene expression. One-day lesioned retinal explants were kept in culture for 4 hours with PBS or GJ blockers. After this period, retinas were processed for quantitative real-time PCR. As shown in Figure 9, we observed downregulation of *caspase 3* gene expression in retinal explants cultured with quinine (−69%, *P*<0.01) and CBX + quinine (−73%, *P*<0.01). *Caspase 8* expression was downregulated after CBX (−54%, *P*<0.05), quinine (−58%, *P*<0.05) and CBX + quinine (−63%, *P*<0.05) treatment. Similar to *caspase 3*, *caspase 9* gene expression was downregulated only after quinine (−63%, *P*<0.01) and CBX + quinine treatment (−64%, *P*<0.01). These results revealed that treatment with GJ blockers regulate the expression of both initial and effector caspases, but in a different, specific manner. Indeed, CBX treatment affected *caspase 8* expression, whereas quinine caused downregulation of all three caspases tested.

### Intraocular Injection of GJ Blockers Affects Apoptosis Spread

Since our results indicated that in vitro treatment with GJ blockers increase cell viability in lesioned retinas, we investigated whether these blockers affect distribution of apoptotic cells in vivo. For these experiments, induction of mechanical trauma was immediately followed by injection of PBS or GJ blockers. After 24 hours, animals were euthanized and retinal transverse sections were obtained for TUNEL labeling.

As a general result, distribution of apoptotic cells changes in retinas treated with GJ blockers. Distribution of apoptotic nuclei was typically narrower in retinas incubated with GJ blockers (Fig. 10, A–D). Once again, we employed two different quantification methods: i) the number of TUNEL-positive nuclei counted from different distances from the lesion focus (Fig. 10, E–H) and ii) normalized values from bitmap analyses (Fig. 10, I–L).

After applying linear regression on experimental data, considering the number of apoptotic cells (Fig. 10M), the absolute value for *a* angular coefficient was higher for quinine 500 µM (−1.55±0.12, P<0.05) and CBX + quinine 500 µM (−1.63±0.18, P<0.05), when compared to PBS (−0.71±0.11). Values obtained from bitmap pixel analysis (Fig. 10N) indicated that the decrease of normalized pixel brightness is larger when eyes were injected with quinine 500 µM (−22.56±3.03, P<0.05) when compared to PBS (−11.98±2.04). Taken together, these results revealed that apoptosis in the retina after mechanical trauma is affected by intraocular injections of GJ blockers.

## Discussion

Mechanical trauma in the central nervous system results in primary cell loss, which is followed by secondary death of neurons. This follow-up event can promote a greater loss of tissue than that caused by the primary insult [17]. Participation of GJ in secondary cell loss, a phenomenon also known as bystander cell death, has been examined in several pathologies, including cancer [18]. However, involvement of GJ communication in apoptosis spread can be solely investigated following some premises. First, cells have to show extensive, effective coupling; second, Cx expression must be demonstrated in dying cells; third, it is imperative that the model generates concentration gradients between cells, driving the exchange of molecules by GJ.

Retinal cells are extensively coupled by GJ channels, forming extensive and reconfigurable networks [9], [19], [20]. Indeed, GJ permeability and Cx expression can be modulated in several physiological conditions, notably during retinal development [5], [21] but also in response to light/dark adaptation [10]. In the first part of this study, we determined that *Cx36* and *Cx43* gene expression levels were respectively maintained and upregulated. Immunohistochemical analysis revealed that, in spite of the specific changes in spatial distribution of both proteins, Cx labeling could be observed in the focus and penumbra areas. Notably, Cx36 were observed close to TUNEL-positive nuclei, revealing the presence of this protein in apoptotic cells or their vicinity. Indeed, triple-labeling experiments confirmed that Cx36 is present in apoptotic neurons.

The functional role of GJ was assessed employing pharmacological blockers, raising usual issues such as cell toxicity and action specificity. CBX, a broad-spectrum GJ blocker, has been used in retinal studies, where no significant toxic effects were reported [22], [23]. Regardless this information, our results indicated that CBX application increased cell viability in vitro, lessening chances of CBX noxious effects. Nevertheless, subjects on specificity of CBX on GJ could be raised. CBX is a synthetic derivative of glycyrrhetinic acid, a pentacyclic triterpenoid with several pharmacological properties, including antiviral, antifungal, antiprotozoal and antibacterial activities. Moreover, it has been reported that CBX may also act on voltage-gated calcium channels in the retina [24]. In order to approach this issue, we have employed a different class of molecule that also acts on Cx-mediated communication. Quinine, an alkaloid that specifically blocks Cx36- and Cx50-channels, changes GJ permeability by distinct mechanisms [25]. Application of quinine to lesioned retinas indicated that blocking of GJ channels, most probably formed by Cx36, increased neuronal survival. Actually, protective effects of quinine started earlier and were also observed when quinine was combined with CBX. Interestingly, previous studies from our group indicated that use of CBX is likely more effective on GJ blocking than quinine, such as during retinal development, when no antiviral, antifungal, antiprotozoal and antibacterial specific activities are on demand [23]. Taken together, these results indicated that GJ blocking properties of alkaloid quinine can be quite useful, although most likely in specific, distinct situations, such as indisputably demonstrated on acute neurodegeneration.

Since we were able to observe that the use of GJ blockers decreased apoptosis spread in an acute trauma model, it could be worth to identify apoptotic molecules which were affected by those blockers. In general, several mechanisms for secondary degeneration have been proposed, including release of free oxygen radicals, alteration of extracellular ion concentrations, high levels of glutamate and altered intracellular calcium [26], [27], [28]. Regarding cell-cell direct communication, calcium concentration seems particularly important, since it is classically associated to apoptosis in the nervous system, plus the fact that fast alterations on intracellular calcium caused by GJ communication were clearly demonstrated in the retina [29], [30]. Furthermore, the GJ transfer of second messengers easily diffusible by these channels, such as IP3, could control calcium cytosolic concentrations based on release by internal stores [31], [32].

Finally, changes in microRNAs pools could also be involved in neurodegeneration and apoptosis spread [33], [34], since the passage of these short molecules (around 21 nucleotides) by GJ channels was recently verified in neural cells [35]. Differences in the biogenesis and degradation of these non-coding RNAs among dying and viable cells may create gradients that trigger the passage of specific microRNA through GJ channels [36]. Indeed, changes in the expression of specific miRNAs may induce apoptosis by distinct mechanisms, including the caspase-dependent pathway [37]. Although speculative, this hypothesis is highly compatible with the results presented in this study.
